# The benefits of ablation using TactiFlex compared with TactiCath in an ex vivo model: a face-to-face experimental comparison study

**DOI:** 10.1007/s10840-024-01897-6

**Published:** 2024-08-20

**Authors:** Yodo Odake, Takehito Tokuyama, Kazutaka Aonuma, Kenji Kuroki, Yasuya Inden, Motoki Furutani, Yousaku Okubo, Sho Okamura, Shunsuke Miyauchi, Shogo Miyamoto, Naoto Oguri, Yukimi Uotani, Takumi Sakai, Yukiko Nakano

**Affiliations:** 1 Department of Cardiovascular Medicine, Graduate School of Biomedical and Health Sciences, Hiroshima University, 1-2-3 Kasumi, Minami-Ku, Hiroshima, 734-8551 Japan; 2 Department of Cardiology, Faculty of Medicine, University of Tsukuba, Tsukuba, Japan; 3 Department of Cardiology, Faculty of Medicine, University of Yamanashi, Chuo, Yamanashi Japan; 4 Department of Cardiology, Nagoya University Graduate School of Medicine, Nagoya, Japan

**Keywords:** Ablation lesion, Catheter ablation, Steam pop, TactiFlex, TactiCath

## Abstract

**Background:**

TactiFlex is a next-generation catheter that is being used increasingly in ablation-treatment strategies. The purpose of this study was to investigate the differences in ablation lesions when the ablation power, time, and perfusion flow are varied with TactiFlex and TactiCath catheters.

**Methods:**

The TactiFlex and TactiCath catheters were contacted perpendicularly/obliquely/parallel to the swine myocardium at varying powers (30, 40, and 50 W), time points (10, 15, 20, 25, 30, and 40 s), and forces (5, 10, 15, 20, and 30 g); the depth, width, and area of each lesion were measured, and the number of steam pops that occurred was counted.

**Results:**

A total of 672 (336 lesions for each catheter) radiofrequency (RF) energy applications were delivered and 648 lesions were analyzed, excluding steam pops. The surface area and volume increased significantly with an increasing contact force for the TactiCath. The TactiCath lesions were significantly deeper than those for TactiFlex in most groups with the same power and seconds. The surface area was significantly larger when the catheters were contacted obliquely/parallel to the myocardium than perpendicularly using TactiCath, whereas the difference was less significant in the case of TactiFlex. In a 10-g contact force ablation experiment, TactiFlex did not cause pops, but TactiCath caused pops in 9.8% of cases.

**Conclusion:**

The TactiFlex lesions were shallower than those of TactiCath. However, TactiFlex catheters reduced the steam pops during RF applications. Those data should help clinicians understand the characteristics of the catheters and develop adequate strategies.

**Graphical abstract:**

**Figure:**
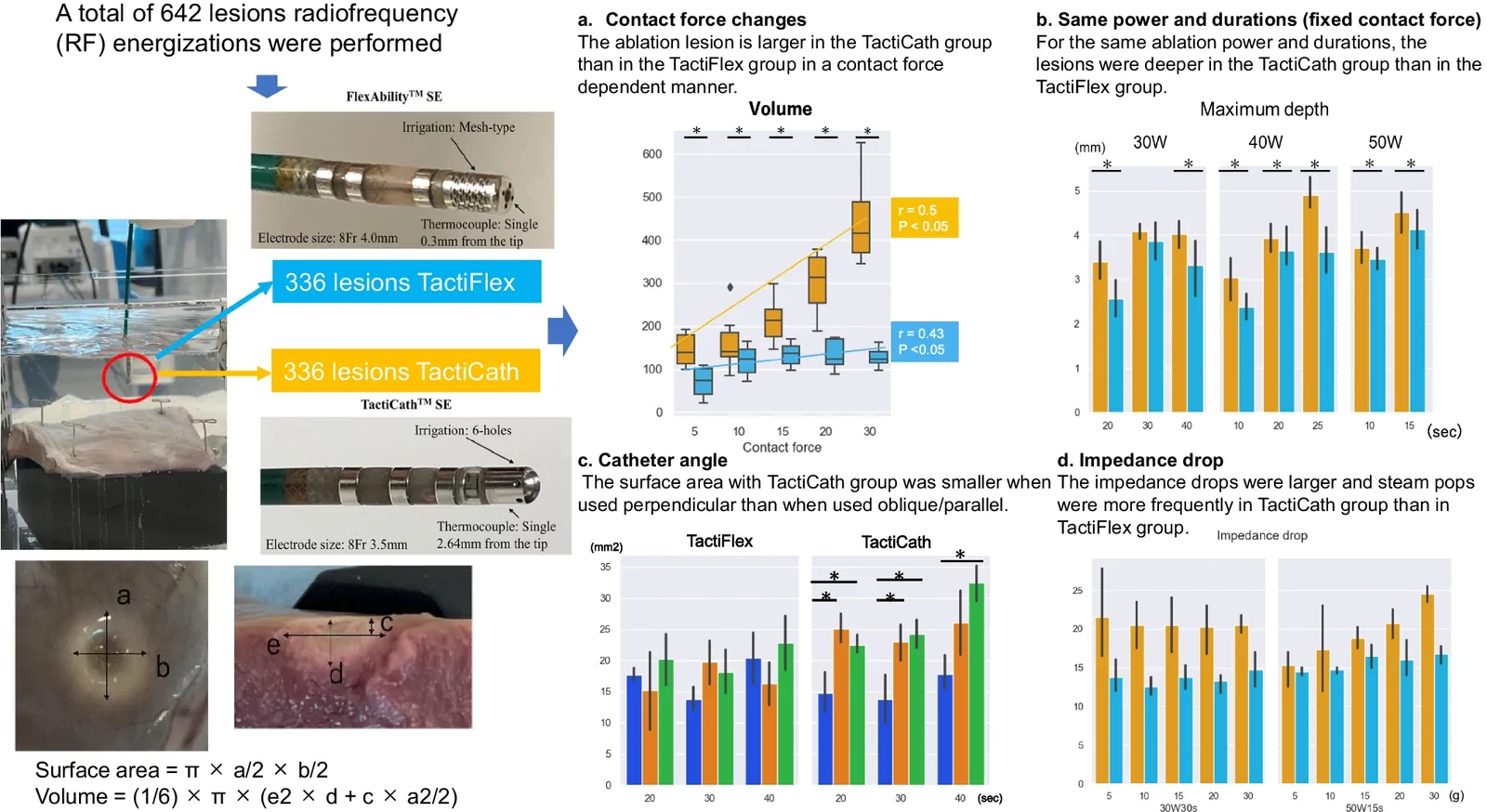

**Supplementary Information:**

The online version contains supplementary material available at https://doi.org/10.1007/s10840-024-01897-6.

## Introduction

Radiofrequency ablation (RFA) is an important curative treatment modality for cardiac arrhythmias and is considered a main therapy for atrial fibrillation. Successful ablation requires the avoidance of excessive heating and an optimal lesion formation [1]. The development of new catheters, such as open irrigation catheters and force sensing catheters, has improved the outcomes and safety of ablation.

Recently, the TactiFlex™ Ablation Catheter, Sensor Enabled™ has become available in the clinical setting. The TactiFlex catheter is a next-generation open-irrigated ablation catheter that combines the force-sensing technology of the TactiCath™ contact force ablation catheter and Sensor Enabled™ catheter with a handle and treatment circuit similar to the FlexAbility™ ablation catheter [2]. The purpose of this study was to investigate the differences in ablation lesions when the ablation power, time, and perfusion flow were varied with the TactiFlex and TactiCath catheters.

**Table 1:** A: Number of steam-pops for each catheter at 30 W/30 s, 40 W/20 s, and 50 W/15 s. B: number of steam-pops for each catheter at 10 g CF

A																
	30 W					40 W					50 W					
	5 g	10 g	15 g	20 g	30 g	5 g	10 g	15 g	20 g	30 g	5 g	10 g	15 g	20 g	30 g	Total
TactiFlex	0	0	0	0	0	0	0	0	0	0	0	0	0	0	0	0
TactiCath	0	0	0	0	0	1 (12.5%)	4 (50%)	5 (62.5%)	37.5%)	0	1 (12.5%)	2 (25%)	1 (12.5%)	2 (25%)	4 (25%)	23 (19.2%)
B																
	30 W			40 W			50 W		Total							
	20 s	30 s	40 s	10 s	20 s	25 s	10 s	15 s	0							
TactiFlex	0	0	0	0	0	0	0	0	0							
TactiCath	0	0	1 (7.1%)	1 (7.1%)	4 (28.6%)	3 (21.4%)	0	2 (12.5%)	11 (9.8%)							

## Methods

### Experimental model

**Fig. 1:**
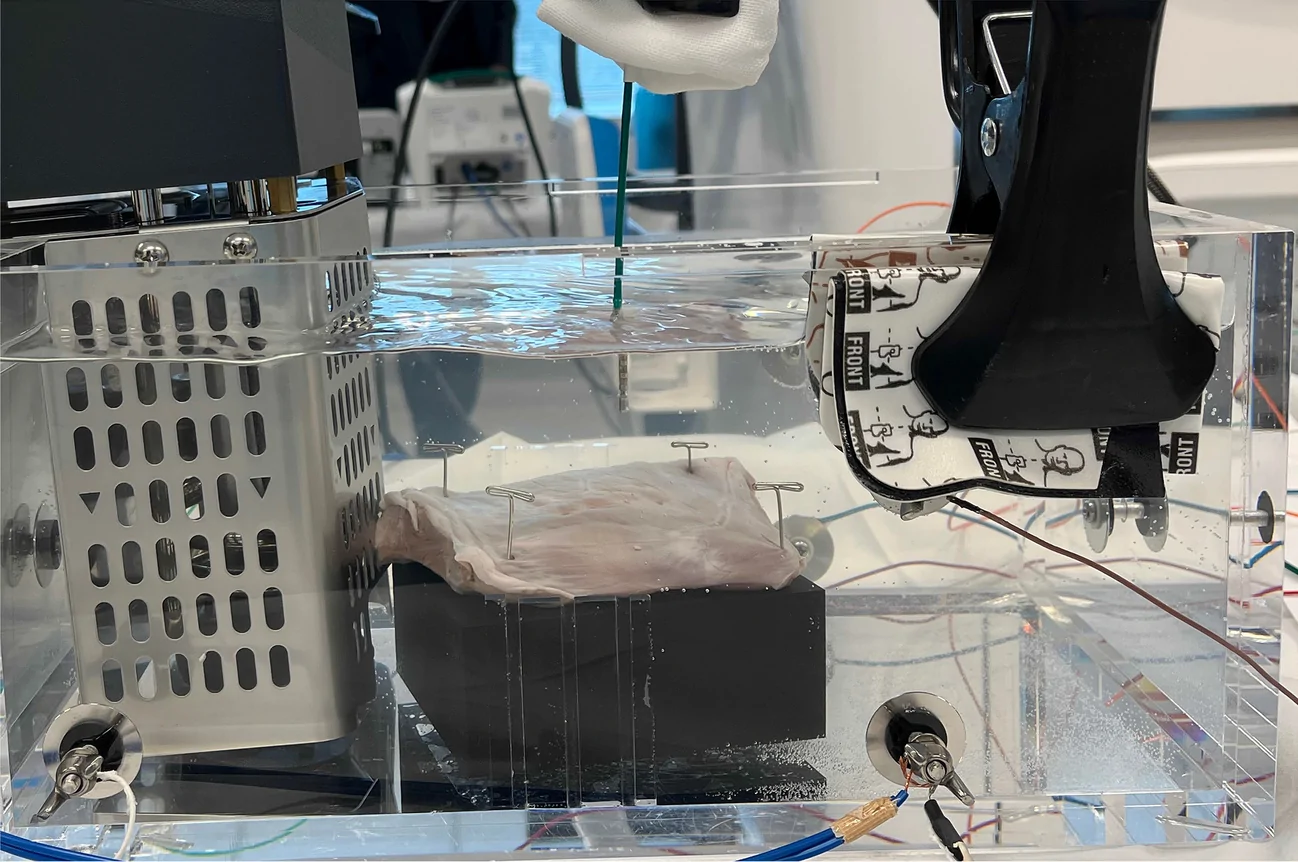
Ex vivo experimental model. This shows an ex vivo model using swine heart tissue in a 37 °C saline bath with a load cell and ablation catheter. Sodium chloride was used to maintain an impedance of 110–120 Ω

An ex vivo model consisting of swine myocardium, a circulating saline bath at 37 °C, a submersible load cell, and an ablation catheter was assembled (Fig. 1). The saline solution was circulated in the saline bath at a constant rate. Sodium chloride was added to the bath to maintain an impedance of 110–120 Ω. As a reflux model, the swine myocardium was placed on a submerged load cell in a circulating saline bath. This study obtained approval from the Hiroshima University Ethics Committee (approval number: Cr2023-0004).

### Ablation protocol

In an experiment to see the difference in the ablation lesions created by the two catheters using contact force, an open-irrigated catheter was positioned perpendicular to the myocardium with an equal force (5, 10, 15, 20, and 30 g) using a fixation device. RFA was delivered at a power of 30, 40, and 50 W with varying time intervals (10, 15, 20, 25, 30, and 40 s). The irrigation flow rate was 13 mL/min for TactiFlex and 17–30 mL/min (17 mL/min at 30 W and 30 mL/min at 40 and 50 W) for TactiCath. In an experiment to see the difference in the ablation lesions between the two catheters depending on the angle of the application, the catheter was placed perpendicularly, parallel, and obliquely (45°) to the myocardium. The incidence of audible steam pops was recorded.

### Ablation lesion analysis

**Fig. 2:**
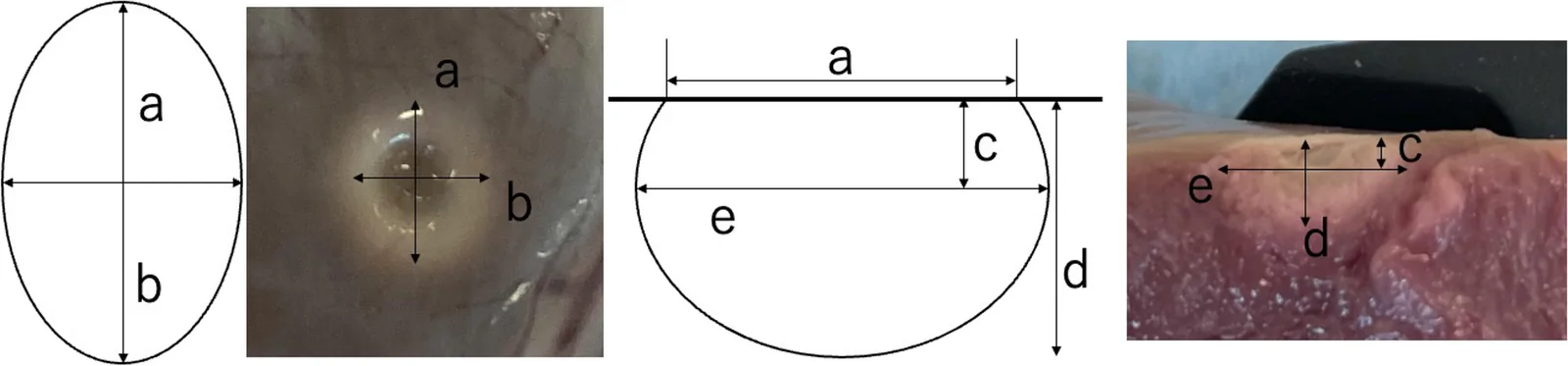
Ablation lesion measurements. For each lesion, the superficial lesion length (**a**), superficial lesion width (**b**), depth at the maximum length (**c**), maximum depth (**d**), and maximum length (**e**) were measured. The surface area and lesion volume were calculated using the following formulas: Surface area = π × a/2 × b/2, Volume = (1/6) × π × (e^2^ × d + c × a^2^/2)

We cut the visual center of the long axis of the lesions as viewed from the surface prior to the experiment. After cutting the tissue to identify the fragments, the characteristics and volume of the ablation lesions were analyzed. The tissues were analyzed using a digital micrometer after the RF delivery. Photographs of representative surfaces and cross-sections of the lesions are shown in Fig. 2. We measured the lesion as shown in Fig. 2. The volumes were calculated using a formula described and validated in a previous study [3].

$$\begin{array}{l}\text{Surface area}=\uppi \times a/2\times b/2\\ \mathrm{Volume}=\left(1/6\right)\times \pi \times \left({e}^{2}\times d+c\times {a}^{2}/2\right)\end{array}$$

Lesions with steam pops were excluded from the measurements.

### Statistical analysis

**Fig. 3:**
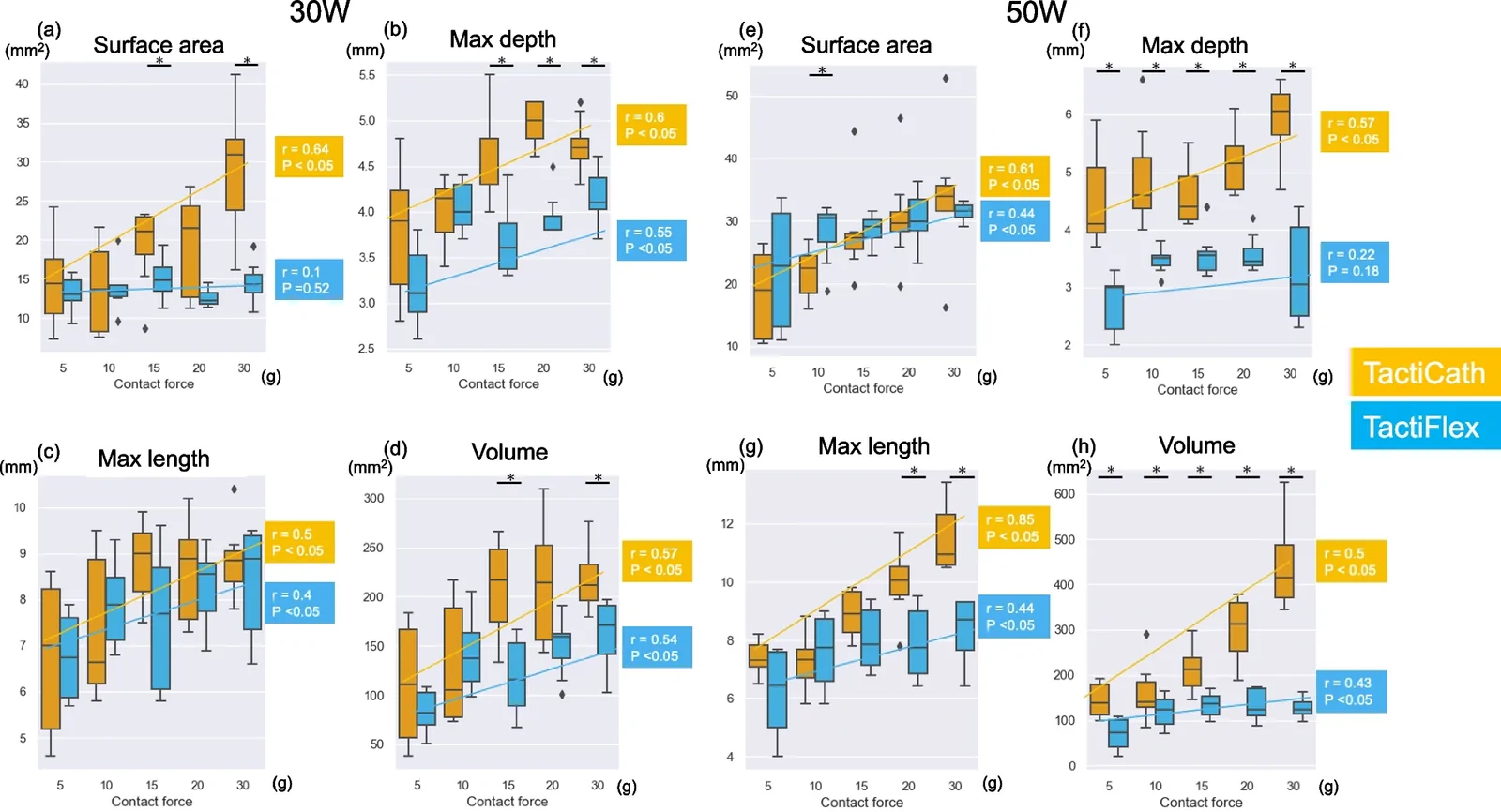
Ablation lesion characteristics after energy applied with 30 W/30 s and 50 W/15 s. The surface area, maximum depth, maximum length, and volume increased significantly with an increasing contact force for the TactiCath, and the difference due to the contact force was more pronounced than with the TactiFlex. Significant differences in the correlation coefficient (*r*) are indicated by the *p*-value. *P < 0.05

The Wilcoxon rank-sum test and the Kruskal–Wallis test were used for group comparisons of continuous variables. In Fig. 3, we used a Pearson’s test and the correlation coefficients (*r*) between the contact force and calculated the surface area, maximum depth, maximum area, and volume. In Fig. 5, the Steel–Dwass test was performed as a subsequent multiple comparison and the results were added. Statistical significance was set at a *P* value of < 0.05.

Data were calculated as the mean ± standard deviation (SD). All statistical analyses were conducted using JMP software, version 15.0 (SAS Institute).

## Results

### Contact force control

A total of 672 RF applications were delivered (336 lesions for each catheter orientation) and 24 lesions were excluded because ablation with TactiCath caused steam pops). Finally, the results of 648 applications were used in the analysis.

The difference in the ablation lesions between the TactiCath group and TactiFlex group with different contact forces is shown in Supplementary Table 1. Figure 3 shows the ablation lesion characteristics, with the ablation applied using each catheter with 30 W/30 s and 50 W/15 s in five groups with contact force (CF, 5–30 g). The surface area, maximum depth, maximum length, and volume increased significantly with an increasing contact force in the TactiCath group, and the difference due to the contact force was more pronounced than in the TactiFlex group. Ablation using TactiCath caused steam pops in 23 cases (23/120 [19.17%]) when ablated with 40 W or 50 W, whereas ablation using TactiFlex did not cause steam pops (Table 1, A).

### Fixed contact force

**Fig. 4:**
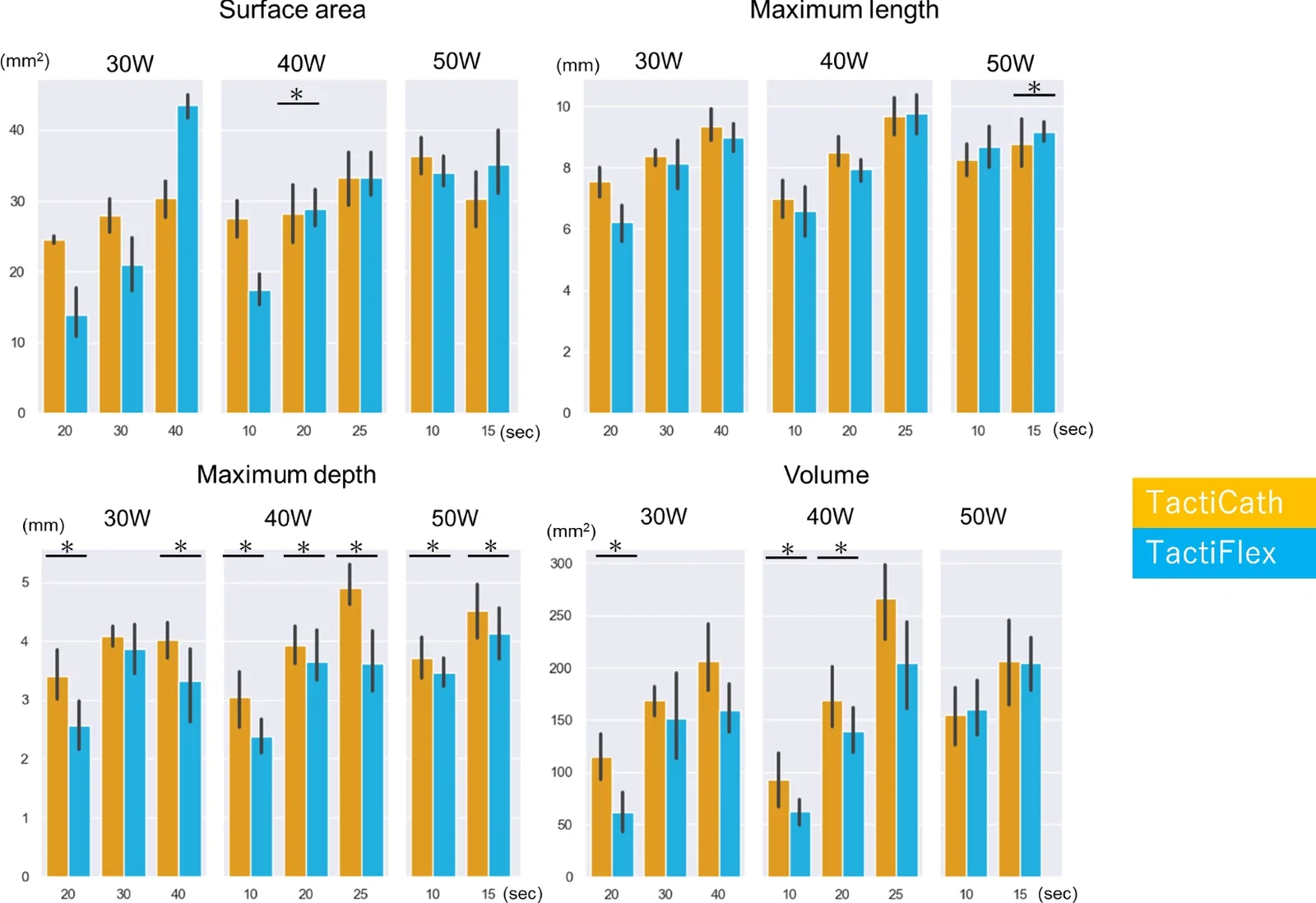
Ablation lesion characteristics for each power and time setting. TactiCath had significantly deeper or tended to have deeper lesions than those of TactiFlex in most groups for the same ablation power and duration. The asterisks (*) indicate a significant difference

Figure 4 shows the ablation lesions generated with 30, 40, and 50 W for the various durations with a CF of 10 g. The results show that the lesions created with TactiCath were significantly deeper than those for TactiFlex in most groups for the same ablation power and duration. For the surface area and maximum depth, the results were inconsistent. As for the volume, the lesions created with TactiCath were larger than those with TactiFlex at 30 W 20 s and 40 W 2 0 s ablation. Ablation using TactiFlex did not cause steam pops, whereas ablation with TactiCath caused steam pops in 11 cases (9.8%]) (Table 1, B).

### Catheter angled lesion

**Fig. 5:**
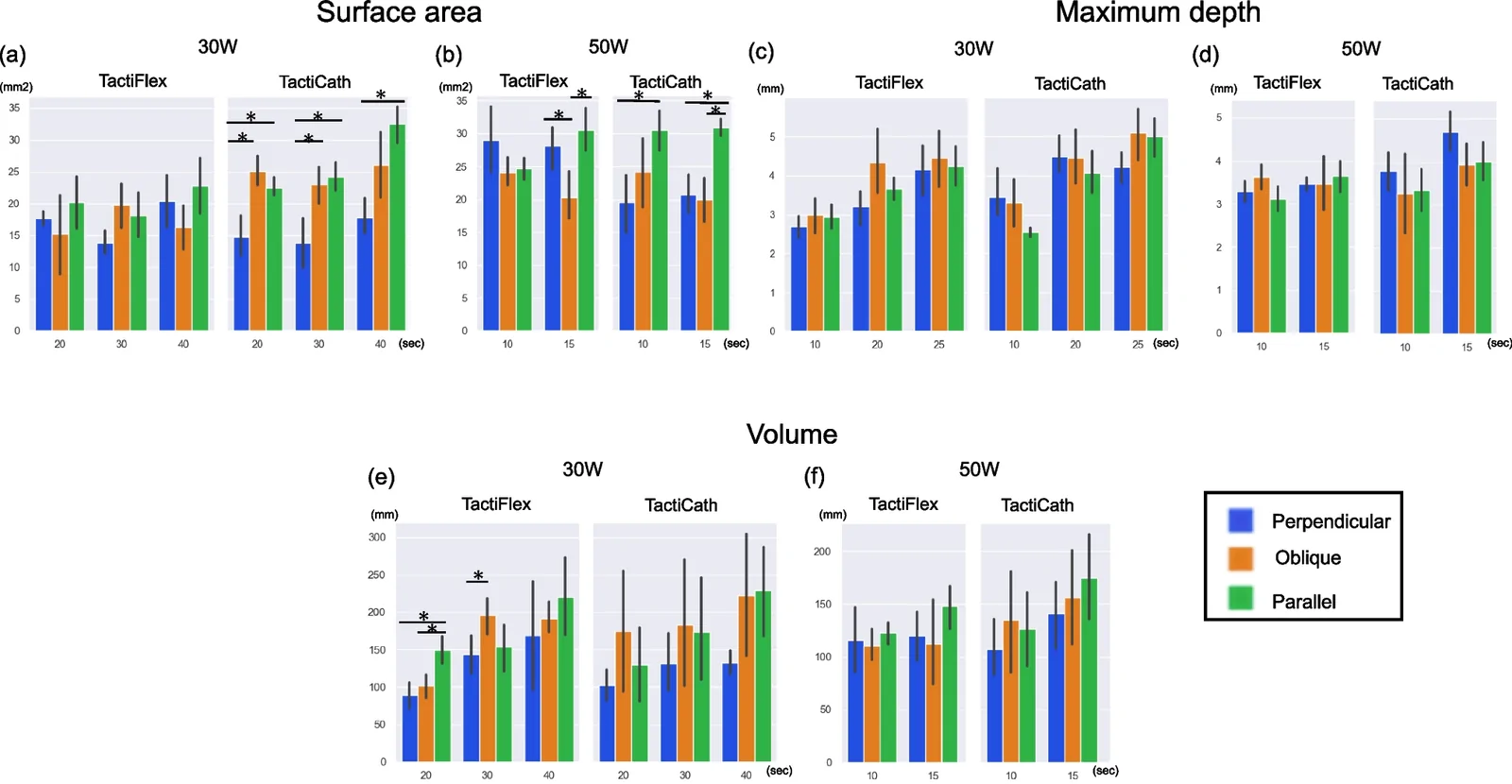
This shows the lesion size obtained after the applications with the catheters at different angles. In the case of TactiCath, the surface area was significantly smaller for the perpendicular application than that for the oblique/parallel applications, but for TactiFlex the difference was not evident

Figure 5 shows the measured lesion dimension for each catheter direction. In the case of TactiCath, the surface area was significantly smaller for the perpendicular application than the oblique/parallel applications, but the difference was not evident for TactiFlex. There were no significant differences in the maximum depth and volume according to the catheter application.

### Impedance drops

**Fig. 6:**
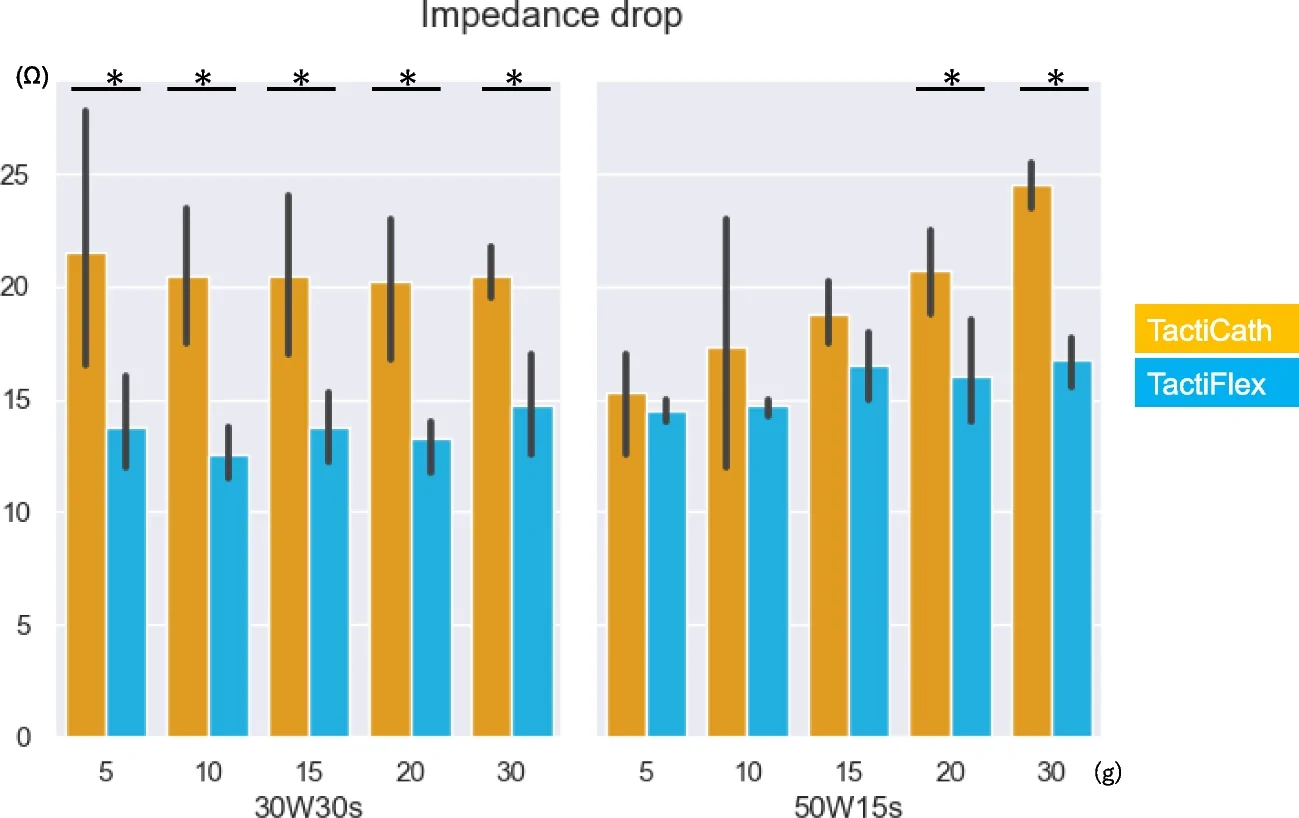
This shows the degree of the impedance drop during ablation at each setting. The impedance drop of TactiFlex was significantly lower than that of TactiCath. The asterisks (*) indicate a significant difference

For TactiFlex and TactiCath, the degree of the impedance drop during ablation with each setting is shown as an impedance drop (Fig. 6). The impedance drop with TactiFlex was significantly lower than that with TactiCath. This difference in the impedance drop was consistent with the large number of steam pops observed in the ablation experiments with TactiCath.

## Discussion

To our knowledge, this was the first study to compare the lesions generated by ablation using the new open-irrigated ablation catheter, TactiFlex, with the lesions obtained using the traditional catheter, TactiCath. This ex vivo study revealed the following points. (1) The surface area, maximum depth, maximum length, and volume increased significantly with an increasing contact force for TactiCath, and the difference due to the contact force was more pronounced than that for TactiFlex. (2) For the same ablation power and duration, the lesions tended to be significantly deeper in the TactiCath group than TactiFlex group. (3) The surface area with TactiCath was significantly smaller when used perpendicular than when used oblique/parallel, but the difference was not apparent with TactiFlex. (4) Impedance drops were larger and pops were more frequent with TactiCath than TactiFlex.

We found that the ablation lesions obtained with TactiCath were deeper than those with TactiFlex. It has been known that for the same contact force, the longer the catheter tip, the greater the area of the catheter tip exposed to saline solution and the greater the cooling effect [4]. The lesions were smaller for the TactiFlex because of its longer tip length (TactiFlex tip, 4.0 mm; TactiCath tip, 3.5 mm). TactiFlex had a lower irrigation flow rate than TactiCath. As the flow rate increased, the resistance of the cathode tip decreased, and as the resistive energy decreased, the conduction energy increased, allowing the energy to reach deeper. In this study, TactiCath had a greater impedance drop than TactiFlex. Because the variables that determine resistance heating are the applied power and impedance [5], TactiCath had greater resistive heating and deeper lesions. Additionally, the differences in the catheter tip morphology may have contributed to the differences in the ablation lesions obtained using the two catheters. The tip of the TactiCath catheter is spherical, whereas the flex tip is cylindrical, which might also have affected the size of the lesion. For the spherical tip, if the CF was small, only a small area of the tip was in contact with the tissue. Another difference in the lesion size between the TactiFlex and TactiCath could be attributed to the different cooling effects on the outside of the TactiFlex catheter due to its mesh structure. Therefore, the surface area and volume of the lesions will be small, and their size will depend on the CF. In contrast, with the cylindrical tip, the entire base is in contact with the tissue, and CF variation has only a slight effect [6, 7]. In this study, TactiFlex yielded little changes in the surface diameter or volume as the CF changed, suggesting that the tip geometry contributed to this result.

Although there were no significant differences in this study, in general, the parallel placement of the catheter is considered to increase the volume because of the larger contact surface with the tissue [8]. The small lesions obtained in the oblique and parallel applications of the catheter may have been due to the fact that the perfusion holes are closer to the tissue compared with the perpendicular application, which distributes to the force on the tissue and cools the surface.

Compared with TactiCath, the TactiFlex catheter had a significantly reduced incidence of steam pops. The TactiCath design, which has a spherical tip, may have provided a larger surface area in contact with the tissue and increased the risk of steam pops. This risk of steam pops is likely even higher with a greater applied CF due to the further increase in the surface area in contact with tissue. Other studies [6, 9] compared various catheter tip designs and found that catheters with spherical tips yielded more steam pops. TactiFlex has 56 irrigation holes and TactiCath has six irrigation holes. The increased number of irrigation holes may also directly increase the cooling effect on the electrode cooling, resulting in fewer steam pops [10]. The trend toward a lower impedance also supports the fact that the ablation lesion depth of TactiFlex is shallower than that of TactiCath and that there are fewer steam pops.

### Study limitations

The first limitation of this study was that it was conducted in an ex vivo model; therefore, caution should be exercised when translating the results directly to clinical practice. The statistical significance of the results varied. This may be due to the limited number of ablation experiments, the fact that the same myocardium was not ablated, and the influence of the differences in the myocardial properties. Furthermore, the characteristics of the lesions in this study may also have differed from those in the clinical setting. For example, the risk of steam pops in ex vivo models did not reflect that of real lesions because of the low tissue viability and the low susceptibility to tissue boiling [11]. A clinical trial of the TactiCath catheter reported a lower steam-pop rate (0.13%) compared with this study [12]. Although we do not believe that these findings are parallel to the actual clinical practice, they may be useful in the future when further verifications are made.

### Clinical implications

Although the findings of this study may not replicate those of the beating heart in the ex vivo study, perhaps it is expected that the comparative differences between the catheters would be relatively similar. A precise understanding of the dimensions of the lesions is an essential parameter in the ablation-treatment strategy. The present study suggested the possibility of improving the success rate of ablation therapy by identifying the differences in the characteristics of the two catheters. In this study, the TactiFlex catheter provided a safer RF application with no steam pops, in addition to having a thermocouple at the distal tip end to accurately monitor the tissue temperature. High-power short-duration ablation performed with TactiFlex, which had fewer steam pops, has been reported to be more effective than conventional low-power ablation with TactiCath, with a shorter procedure time [13, 14]. In turn, TactiCath produced more steam pops but may be efficient when the lesion depth is large and requires ablation on the left ventricular wall or deeper lesions. However, the data generated in this study cannot be directly applied to the clinical setting of the beating human heart, especially with respect to the lesion size. Nevertheless, these data should assist clinicians in performing this intervention, to understand the relationships among the parameters, and to plan a strategy of experimentation accordingly.

## Conclusions

For the same ablation power and time, the ablation lesions were significantly deeper in the TactiCath group than TactiFlex group. The steam pop frequency was greater in the TactiCath group than TactiFlex group. Ablation using TactiFlex was less sensitive to the catheter angle and contact force than that using TactiCath.

## Supplementary Information

Below is the link to the electronic supplementary material.

Supplementary file1 (DOCX 24 KB)

## Data Availability

The data that support the findings of this study are available from the corresponding author, [T.T.], upon reasonable request.
