# Supplementary material for: The benefits of ablation using TactiFlex compared with TactiCath in an ex vivo model: a face-to-face experimental comparison study
Source: J Interv Card Electrophysiol. 2024 Aug 20;69(7):1661–8. doi: 10.1007/s10840-024-01897-6 (PMC13687357; doi:10.1007/s10840-024-01897-6)
Supplement: Supplementary file 1 [file 10840_2024_1897_MOESM1_ESM.docx]

Supplementary Table.1

Ablation lesion characteristics after energy applied at 30 W/30 sec, 40 W/20sec and 50 W/15 sec. Significant differences in the correlation coefficient (r) were indicated by p value.
